# The Effect of Ethanol on the Compound Thresholds and Aroma Perception in Chinese Baijiu

**DOI:** 10.3390/molecules30040933

**Published:** 2025-02-17

**Authors:** Jialing Lu, Jia Zheng, Dong Zhao, Yan Xu, Shuang Chen

**Affiliations:** 1 Key Laboratory of Industrial Biotechnology of Ministry of Education, School of Biotechnology, Jiangnan University, Wuxi 214122, China; lujialingptj@163.com (J.L.); yxu@jiangnan.edu.cn (Y.X.); 2 Flavor Science Innovation Center, Technology Research Center, Wuliangye Yibin Co., Ltd., Yibin 644000, China; zhengwanqi86@163.com (J.Z.); zhaodong@wuliangye.com.cn (D.Z.)

**Keywords:** ethanol, threshold, aroma attribute, Baijiu

## Abstract

Strong-aroma type Baijiu and its diluted samples were characterized through descriptive analysis. Significant changes were observed in four (ethanol, Jiao-aroma, fruity, and grain) of the nine aroma attributes, primarily attributed to variations in ethanol concentration rather than other compounds. The thresholds of 40 compounds in aqueous solutions with alcohol concentrations of 20%, 30%, 40%, 50%, and 60% were measured using three-alternative forced-choice tests. The thresholds of 30 compounds were significantly positively correlated with ethanol concentration. The thresholds of 40 compounds were affected by ethanol concentration to varying degrees, with changes ranging from 2 to 692 times. Due to the varying degrees of alcohol influence on the compound threshold, the aroma profile of diluted Baijiu sample is different from the original Baijiu sample.

## 1. Introduction

Ethanol is the most abundant flavor compound in alcoholic beverages, particularly in distilled spirits. Chinese Baijiu, a type of distilled spirit, typically has an ethanol concentration of 50–60% ABV (alcohol by volume) [1,2]. With the change of consumer demand and the development of internationalization of Baijiu, the trend towards lower alcohol content has become a key direction for Chinese Baijiu industry development, resulting in ethanol concentration of Baijiu ranging from 20 to 50% ABV. For example, the 39% ABV strong-aroma type Baijiu from Yibin Wuliangye Co., Ltd. (Yibin, China) has garnered widespread attention and recognition. Previous studies have shown that ethanol concentration plays an important role in shaping the aroma profile of alcoholic beverages [3,4,5,6,7]. Most studies [5,6] have focused on the alcoholic beverages with alcohol by volume lower than 40%, such as beer, wine, and whiskey. To produce low-alcohol Baijiu that appeals to a broader consumer base, it is very important to explore the change of flavor perception during the dilution process of Baijiu.

The release of aroma compounds is influenced by ethanol concentration, which changes the volatilization of these compounds in the headspace [8,9,10,11,12,13,14]. When the concentration of ethanol changes, the structure of the liquid water/ethanol matrix will change, which affects the hydrogen bonding state and the solubility of compounds in the solution, ultimately leading to changes in the volatilization of these compounds [15]. In addition, the pungent smell of ethanol can affect the threshold of the compounds [9,16]. Exploring how ethanol concentration affects the thresholds of aroma compounds is also crucial for gaining a comprehensive understanding of changes in aroma perception. It is well known that an increase in ethanol concentration will lead to a higher threshold for aroma compounds. Guth first proved that the threshold of compounds in ethanol aqueous solution is 10–312 times higher than in water [17]. While the thresholds of compounds have been measured in different ethanol aqueous solutions [18,19,20,21], these threshold data were obtained from different laboratories using different methods. To the best of our knowledge, very few literatures have systematically studied the effect of ethanol concentration on compound thresholds. Additionally, the range of alcohol content studied is quite narrow. Thus, research on the effect of alcohol content on compound threshold remains relatively blank. Due to the effect of ethanol on compound release and threshold, aroma perception is also influenced by the ethanol concentration [5,6,7,22,23,24]. Research exploring the effects of ethanol concentration on the aroma perception of distilled spirits is very important, given the wide range of ethanol concentration, from 40% ABV (usually in the distilled spirits neat) to 20% ABV (commonly found in the diluted distilled spirits before consumption) [7]. However, related research in Baijiu is scarce, and the majority of studies were focused on wine or wine model systems [24,25,26,27].

To sum up, the objectives of this study are: (I) to characterize the aroma profile of the origin Baijiu sample and its diluted samples using DA to investigate the effect of ethanol on flavor perception; and (II) to measure the thresholds of 40 important aroma compounds in aqueous solutions with alcohol concentrations of 20%, 30%, 40%, 50%, and 60% by a three-alternative forced-choice (3-AFC) test to analyze the causes of changes in aroma profiles.

## 2. Results

### 2.1. Effect of Dilution on Sensory Profile of Baijiu Samples

The profile of original Baijiu and diluted Baijiu was studied by descriptive analysis (DA) to characterize the change in aroma perception during diluting. When the Baijiu samples were diluted, both the ethanol content and other substances concentrations changed. To study which factor mainly affects the perception of Baijiu, the sample N-30E was added in the study. N-30E was diluted with an aqueous 61.8% ABV solution using the same dilution ratio as N-30. This means that N-30E and N had the same ethanol content but different other substances concentrations, while N-30E and N had different ethanol contents but the same other substances concentrations.

#### 2.1.1. Assessment of Panel Performance

After discussion by the panel, a total of nine aroma attributes were determined to describe the aroma profile of Baijiu samples, including fruity, ethanol, sweet, jiao-aroma, sour, green, grain, bran, and musty. As shown in Table 1, each aroma attribute was defined. These attributes can not only describe the aroma profile of samples but also reflect the difference in aroma between samples.

In order to ensure the reliability of the result (Table S1), the performance of the panel was assessed for each sensory attribute separately [28]. Thus, as shown in Table 2, an Analysis of Variance (ANOVA) was performed on each sensory attribute. In general, there are three main effects and three interaction effects. The results of dilution sample effect, dilution sample and panelist (D-P) interaction, dilution samples, and session (D-S) interaction are of utmost importance, as they indicate whether the panel can consistently (repeatably) and consensually (agreeably) differentiate the products (discrimination) [29]. With *p* > 0.05, the D-P interaction and D-S interaction are not significant, indicating that there is consensus among the panelists for all aroma attributes, and the panel is repeatable from one session to another. Thus, the panel evaluated the samples consistently and consensually. Based on the repeatability and consistency of the panel, the dilution sample effect of ethanol, Jiao-aroma, fruity, and grain attributes was significant (*p* < 0.05), which means four of the nine aroma attributes were significantly different. Thus, the four attributes were chosen for the further study.

#### 2.1.2. Changes in Aroma Attribute Intensity During Dilution

**Figure 1:**
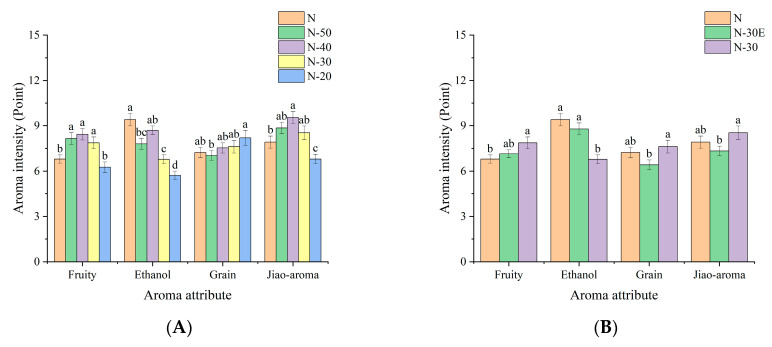
Column chart of the intensity of aroma attribute in N, N-50, N-40, N-30 and N-20 (**A**), and N, N-30E and N-30 (**B**) (a–d: Superscripts of the same letter within an attribute indicate no significant difference by Fisher’s least significant difference (LSD) test at α = 0.05).

Figure 1A,B illustrated the trends in the intensity changes in the four aroma attributes with significant changes. During the dilution process with ultrapure water (Figure 1A), the ethanol aroma is most affected by dilution, while the grain aroma is least affected. As the dilution ratio increases, the intensity of fruity aroma and Jiao-aroma first rises and then decreases. The Jiao-aroma is a characteristic aroma in strong-aroma type Baijiu and is rarely perceived in other alcoholic beverages. Thus, there are currently no studies reporting on the change in its aroma intensity during dilution. While some studies have proved that the fruity attribution is affected by ethanol content [23,25]. But the trend observed here does not fully align with previous research, which shows higher fruity attributes in lower ethanol content solutions. This discrepancy may be due to the previous study being conducted under a different alcohol content (0–20% ABV). In addition, the grain aroma showed an upward trend, while the ethanol aroma exhibited a downward trend.

The samples of N and N-30E contain the same ethanol concentration but differ in the concentration of other substances, while the samples of N-30E and N-30 contain the same concentration of other substances but differ in ethanol concentration. Comparing the three samples (Figure 1B), it can be found that when the ethanol concentration remained constant and the other substances concentrations decreased, the intensity of each aroma attribute did not change significantly. However, when the other substances concentrations remained unchanged and the ethanol concentration decreased, the intensity of the ethanol aroma decreased significantly, while the intensity of the grain aroma and Jiao-aroma increased significantly. This indicated that the change in the concentration of other substances had a lower effect on the intensity of aroma attribute intensity than the change in ethanol concentration. A decrease in ethanol concentration led to a decrease in the compound threshold, thereby enhancing the aroma intensity of aroma compounds. While a decrease in the concentration of other substances reduced the aroma intensity perceived by panelists [30]. The interaction of these two effects resulted in the unpredictable change in aroma attributes. However, because different aroma compounds are affected by the two effects differently, there are also differences in the change of aroma attributes presented by aroma compounds.

**Figure 2:**
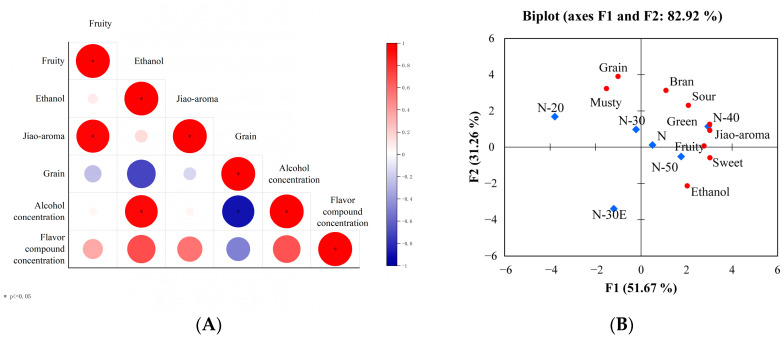
The Spearman correlation heatmap between four aroma attributes, ethanol concentration, and other substances concentration (**A**); The PCA bi-plot generated from the sensory descriptors (**B**).

Correlation analysis was carried out on the four aroma attributes, ethanol concentration, and the concentration of other substances. The results are shown in Figure 2A; ethanol concentration was significantly positively correlated with ethanol aroma and significantly negatively correlated with grain aroma. There was no significant correlation between other substances concentrations and any aroma attribute.

In order to further clarify the difference in aroma characteristics of the samples, principal component analysis (PCA) was conducted as shown in Figure 2B. The first two principal component factors collectively explain 82.92% of the total variance. The aroma profile of samples N-50, N-40, and N-30, diluted with ultrapure water to 50%, 40%, and 30% ABV, is similar to that of the original sample N. In contrast, the aroma profile of sample N-20 was quite different from that of sample N, with a stronger unpleasant smell (musty aroma). Perpète demonstrated that increasing ethanol concentration can reduce unpleasant flavor [3].

**Figure 3:**
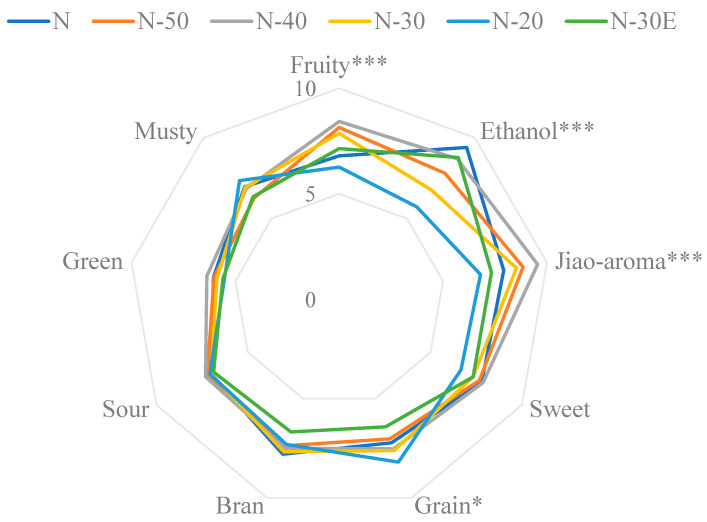
The radar chart of the aroma profile change of Baijiu during dilution. * represent *p* < 0.05; *** represent *p* < 0.001.

A total of four of the nine aroma attributes evaluated changed significantly during dilution, which was mainly caused by the change in ethanol concentration, rather than the concentration of other substances. As shown in Figure 3, the overall aroma profile does not change dramatically, which may explain why strong-aroma style Baijiu is the most popular low-alcohol Baijiu on the market. Because of this, it is crucial to pay attention to aroma properties with significant changes. Because this is a key factor in distinguishing the aroma of high and low alcohol Baijiu. For example, when the strong aroma style Baijiu is diluted and ethanol content is 60% to 50 (*v*/*v*), it is unnecessary to care about all these four aroma contributes, because there are only two attributes (fruity and ethanol aroma) that are significant. Thus, it will reduce the workload when the producer blends the low alcohol finish Baijiu. Based on the change of aroma intensity, the perception of aroma compounds was not equally affected by changes in ethanol concentration. Therefore, in order to elucidate the causes of changes in aroma profile, the next step of this study will be to explore how changes in ethanol concentration affect aroma perception.

### 2.2. Effect of Ethanol on the Aroma Perception

#### 2.2.1. Effect of Ethanol on the Threshold of Aroma Compounds

When the concentration of a compound is constant, the threshold value will affect the aroma contribution of the compound. Flavor compounds exhibit different aroma thresholds in aqueous solutions with different ethanol levels [16,17]. The systematic measurement of compound thresholds in aqueous solutions with different ethanol levels is useful for analyzing the cause of the influence of ethanol concentration on aroma perception.

In this study, a total of 40 compounds with aroma contributions in Baijiu were selected for research. Some of these are skeleton components, such as alcohols, acids, and esters, while others are present in lower concentrations but have very low thresholds, such as lactones, aldehydes, ketones, and sulfur compounds. All of them play an important role in Baijiu’s profile.

**Figure 4:**
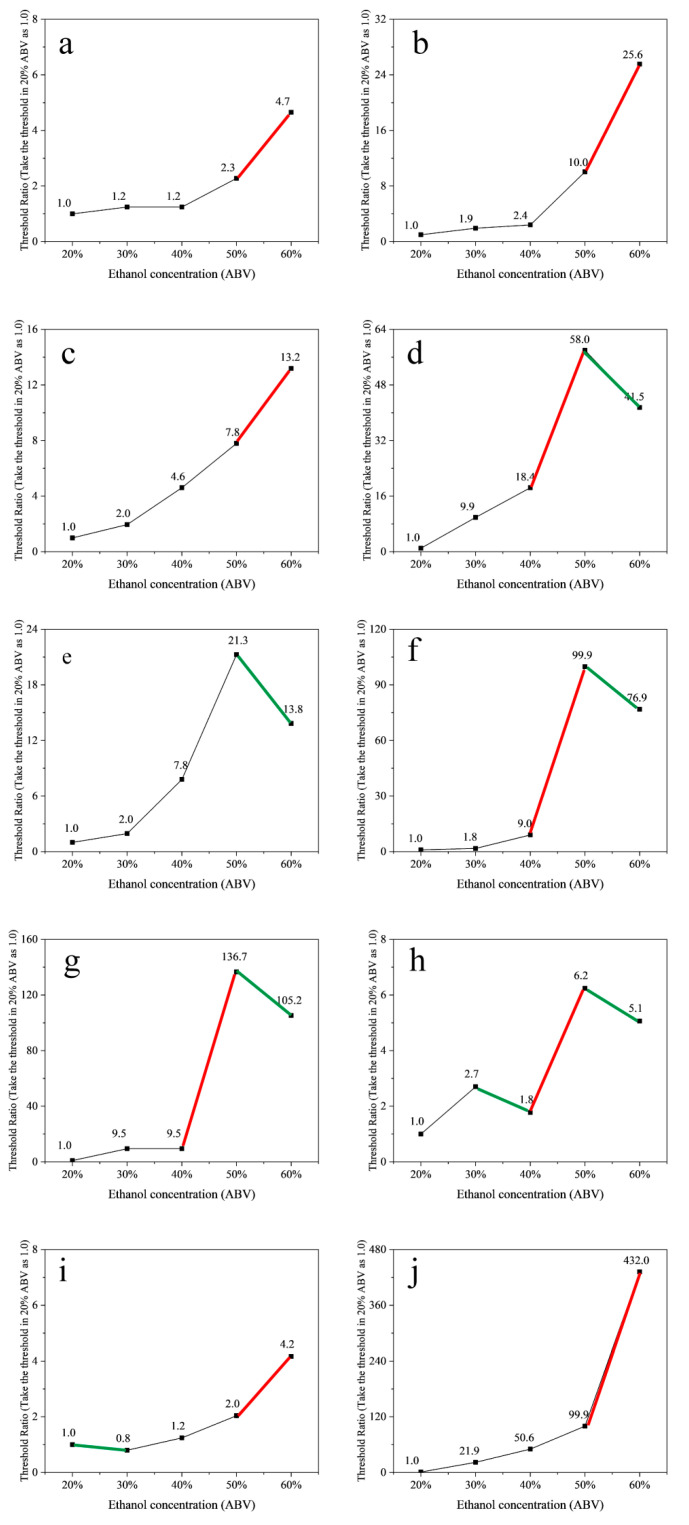
Scatter plots of thresholds ratio in five ethanol levels of ethyl acetate (**a**), ethyl hexanoate (**b**), ethyl heptanoate (**c**), 1-pentanol (**d**), 1-hexanol (**e**), damascenone (**f**), 1-nonanal (**g**), γ-dodecalactone (**h**), dimethyl trisulfide (**i**), and (±)-geosmin (**j**). (The red line presents a downward trend in the threshold when the alcohol content increased, and the green line the interval in which the threshold for each compound is sharply increased).

Following the alcohol content design of DA in 2.1, the thresholds of 40 compounds were determined in aqueous solutions with 60%, 50%, 40%, 30%, and 20% ABV. The result of the threshold was shown in Table 3. The thresholds of compounds generally showed an upward trend as ethanol concentration increased. According to the Pearson correlation analysis, a total of 30 compounds showed a significant positive correlation between the thresholds and ethanol concentration, while 10 compounds showed no significant correlations. These include ethyl acetate, ethyl hexanoate, ethyl heptanoate, 1-pentanol, 1-hexanol, damascenone, 1-nonanal, γ-dodecalactone, dimethyl trisulfide, and (±)-geosmin. The scatter plots of thresholds ratio for these 10 compounds in aqueous solutions at 5 ethanol levels were shown in Figure 4. It is noted that the thresholds of 6 compounds (including 1-pentanol, 1-hexanol, damascenone, 1-nonanal, γ-dodecalactone, and dimethyl trisulfide) showed a downward trend when the ethanol concentration increased, primarily within the 50% to 60% ABV range (indicated by green lines in Figure 4). This may differ from conventional understanding, but the trend was observed in Liu’s study [16]. When the ethanol content increased from 46% to 60% ABV, the thresholds of butyric acid and tetramethylpyrazine decreased. In addition, the thresholds for these 10 compounds increased dramatically in the range from 40% to 50% ABV or from 50% to 60% ABV, as indicated by the red lines in Figure 4.

The esters studied in this study are all straight-chain ethyl ester compounds, which mainly exhibit fruity and floral aromas. At each ethanol concentration level, as the carbon chain length increased, the thresholds of esters initially showed a decreasing trend, followed by an increasing trend (except for ethyl caprylate) (Table 3). Ethyl acetate has the highest threshold value among this series of compounds. The short-chain ethyl ester compound has strong polarity and high solubility in solution, which makes it more difficult to volatilize from the solution. Therefore, its threshold value is high. As the carbon chain length increases, the hydrophobicity of the compound increases, which reduces its solubility and makes it more volatile. Therefore, the threshold shows a downward trend. When the carbon chain continues to increase, the molecular weight and boiling point also increase, making it more difficult to volatilize. Thus, the threshold shows an upward trend [31]. Ethyl valerate has the lowest threshold due to its moderate carbon chain length and molecular weight, as well as its low solubility [28]. When the alcohol content increased from 20% to 60%, the ratio of the maximum threshold to the minimum threshold for ethyl acetate, ethyl propionate, ethyl butyrate, and ethyl heptanoate was smallest, and the ratio was about 5.0. The largest ratio was observed for ethyl hexanoate, with a ratio of 25.6, followed by ethyl nonanoate with a ratio of 20.0. The remaining three compounds have a ratio of about 13.0 (Table S2).

The acids studied in this study are all straight-chain fatty acid compounds, which present off-odors (such as cellar mud, sour, and sweat odor) at high concentration and pleasant aromas (such as fruity and sweet aromas) at low concentration. Similar to straight-chain ethyl ester compounds, acetic acid has the highest threshold, while valeric acid has the lowest threshold at the same alcohol concentration. In addition, the change of the threshold value from acetic acid to heptanoic acid also showed a trend of first decreasing and then increasing. However, the threshold of octanoic acid and decanoic acid showed a decreasing trend, which is worth studying. Butyric acid is the compound whose threshold is least affected by ethanol concentration. The threshold of propionic acid is most affected by the change of ethanol concentration, and the threshold value in aqueous 60% ABV solution is 20.5 times that in aqueous 20% ABV solution (Table S2).

Alcohols are one of the skeleton components in Baijiu. Their thresholds are generally higher than 1 mg/L, mainly showing floral, fruity, and ethanol aromas. While enols, unlike saturated alcohols, have a relatively low threshold of just a few micrograms per liter. Among the eight alcohol compounds measured in Table S2, the compound with the largest threshold change is 1-pentanol, followed by linalool, 1-hexanol, and 2-butanol. Different from other compounds, the thresholds of four compounds, including 1-pentanol, 3-methyl-1-butanol, 1-hexanol, and linalool, were higher in aqueous 50% ABV solutions than in aqueous 60% ABV solutions. This may be due to the high alcohol content benefiting the perception of these four compounds.

In addition to esters, acids, and alcohols, the threshold of 16 compounds with low thresholds was measured, such as aldehydes, ketones, lactones, and sulfur-containing compounds (Table 3). Aldehydes and ketones mainly present green, floral, and fruity. Lactones are mainly present in creamy, coconut, and fruity. And sulfides are mainly peculiar smells. Compared with other compounds at the same concentration, these compounds have a greater contribution to the aroma profile of Baijiu due to their lower thresholds. Therefore, it is of great significance to study the variation law of these compound threshold changes with the change of ethanol concentration.

The thresholds of these 16 compounds were generally more affected by ethanol concentration than the skeleton components. The range of the threshold value of bis-(2-methyl-3-furyl)-disulfide was the largest, and the ratio of the highest threshold to the lowest threshold was 691.8 times, followed by (±)-geosmin of 432.0 times.

Preliminary studies have shown that increasing ethanol concentration enhances pungent odors in alcoholic beverages, making aroma compounds more difficult to perceive [30]. Therefore, the thresholds of compounds tend to rise as ethanol concentration increases. However, by systematically measuring the compound thresholds at different ethanol concentrations in this study, it was found that the thresholds of certain compounds decreased as the ethanol concentration increased. It is speculated that within some specific ethanol concentration ranges, the pungent smell of ethanol may enhance the perception of compounds with the similar aroma.

Different compounds have different threshold changes with the change in ethanol concentration due to their different physicochemical properties and sensory properties. Among the 40 compounds tested, 30 compounds showed a significant positive correlation between the threshold and the ethanol concentration, while 10 compounds had no significant correlations. There were also differences in the degree to which the thresholds of different compounds were affected by ethanol concentration. When the ethanol concentration changed from 20% to 60% ABV, the threshold of butyric acid changed by only 2.3 times, while bis-(2-methyl-3-furyl)-disulfide changed by 691.8 times. Aroma compounds contribute to different aroma attributes, and the change rule of the threshold value of each aroma compound plays an important role in the change of the aroma attributes in Baijiu. Especially for these compounds with significant differences in threshold variation, they may result in significant changes in the aroma profile. Thus, the compounds with huge threshold changes need to be focused on when the strong-aroma style Baijiu is diluted. But it does not mean that the compounds with slight variation need not be focused. The aroma attributions contributed by the compounds with significant differences in threshold can be amplified during the dilution; as a result, the aroma attributions contributed by the compounds with slight differences in threshold can be suppressed during the dilution. And the relationship needs to be further studied; this will be discussed by using OAV (odor activity value).

#### 2.2.2. Effect of Threshold on the Aroma Profile in Baijiu

After the Baijiu sample was diluted, both the concentration of ethanol and other aroma compounds decreased. The decrease in alcohol content enhances aroma perception, while the decrease in aroma compound concentration inhibits aroma perception. As a result, each aroma attribute does not all show a gradient descent trend.

**Figure 5:**
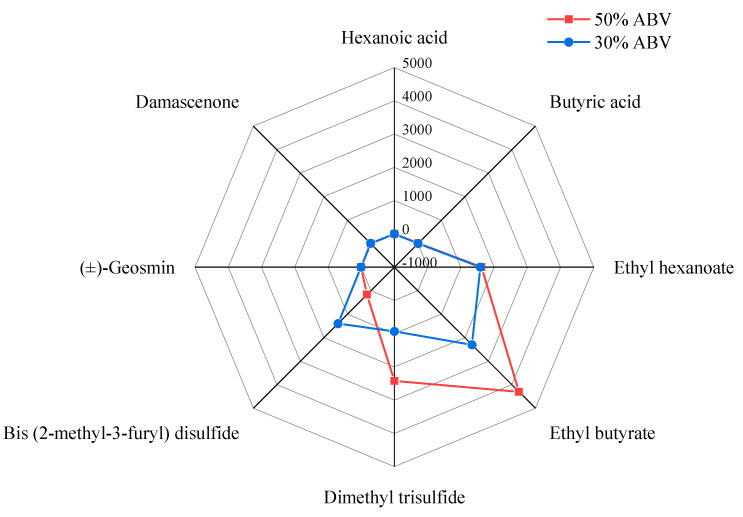
The effect of dilution on OAV.

When the Baijiu was diluted with water, the content of alcohol and aroma compounds changed regularly. However, the thresholds of aroma compounds changed irregularly, which led to the change in the aroma compounds’ OAV changing differently (Table 4). And in order to show the change of compounds aroma contribution, a total of 14 compounds, including ethyl propionate, ethyl heptanoate, ethyl caprylate, ethyl caprate, propionic acid, butyric acid, valeric acid, hexanoic acid, 2-methyl-1-butanol, 1-hexanol, linalool, damascenone, (±)-geosmin, and 4-ethyl-2-methoxyphenol, were used as examples in this study. And their concentrations in Baijiu were referred to previous studies [32,33]. As shown in Table S3, when the Baijiu sample was diluted by ultrapure water from 50% to 30% ABV, the concentration of each compound gradually decreased. However, due to the different properties of each compound, the threshold of each compound is affected by the ethanol concentration to varying degrees, resulting in different changes in the OAV of each compound. The OAV% of each compound was calculated by dividing the OAV of each compound by the total OAV, which can, to some extent, represent the contribution rate of each substance’s aroma. As shown in Figure 5, the compound with the highest aroma contribution in 30% ABV was damascenone instead of ethyl heptanoate in 50% ABV. Additionally, the profile of the aroma contribution changed as well. In the 50% ABV, the aroma contributions of 11 compounds were relatively balanced. However, in the 30% ABV, the aroma contributions of damascenone were extremely higher than others, which were up to 38%. When the Baijiu was diluted with water, the role of compounds in the aroma profile of Baijiu changed, leading to irregular variations in the intensities of aroma attributes in Baijiu.

## 3. Discussion

In this study, a descriptive analysis of strong-aroma type Baijiu sample and its diluted samples was performed. A total of four of the nine aroma attributes evaluated changed significantly, mainly due to changes in ethanol concentration rather than the concentrations of other substance compounds. The 3-AFC method was used to systematically investigate the effect of ethanol concentration (60%, 50%, 40%, 30%, 20% ABV) on the thresholds of 40 compounds. Among them, the threshold of 30 compound threshold was significantly positively correlated with ethanol concentration. Further analysis found that the thresholds of different compounds were affected to different degrees by ethanol concentration, and the ratios of the threshold of a compound in aqueous 60% ABV solution versus aqueous 20% ABV solution ranged from 2 to 692 times. This difference indicates that the aroma contribution of each aroma compound will change during dilution, which may be one of the key factors for the irregular changes in aroma attribute intensity.

## 4. Materials and Methods

### 4.1. Reagents and Standards

All standards of the aroma compounds used in the study were chromatographic grade with a purity of ≥97%. The following compounds were purchased from Sigma Aldrich in Shanghai, China: Ethyl acetate, Ethyl propionate, Ethyl valerate, Ethyl heptanoate, Ethyl caprylate, Ethyl caprate, Acetic acid, Propionic acid, Heptanoic acid, Octanoic acid, Decanoic acid, 2-Butanol, 1-Pentanol, Linalool, 1-Nonanal, β-Lonone, Fema 3377, γ-Dodecalactone, γ-Decalactone, γ-Valerolactone, γ-Octanoic Lactone, γ-Nonanolactone, Furfuryl mercaptan, Bis-(2-methyl-3-furyl)-disulfide And 4-Ethyl-2-Methoxyphenol. The following compounds were obtained from Aladdin in Shanghai, China: Ethyl Butyrate, Ethyl Hexanoate, Butyric Acid, Valeric Acid, Hexanoic Acid, Ethyl Nonanoate, 1-Propanol, 1-Butanol, 2-Methyl-1-Butanol, 3-Methyl-1-Butanol, 1-Hexanol, Damascenone, FEMA 3377, Dimethyl trisulfide, 3-(Methylthio)propionaldehyde, and (±)-Geosmin. Ethanol (High Performance Liquid Chromatography-grade, 99.9%) was purchased from J&K Scientific in Shanghai, China. Water was purified by the Milli-Q water purification system (Millipore, Bedford, MA, USA).

### 4.2. Samples, Model System, and Sensory Reference Preparation

**Figure 6:**
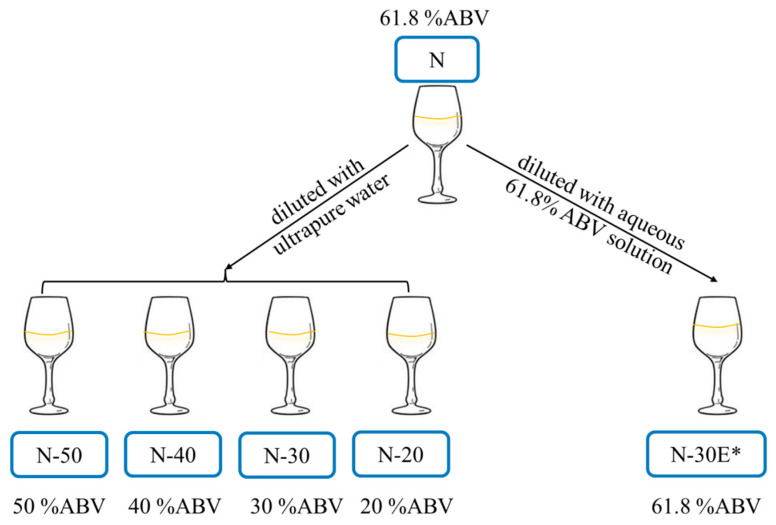
Schematic diagram of Baijiu samples preparation (* N-30E was diluted with aqueous 61.8% ABV solution using the same dilution ratio as N-30).

A commercially available strong-aroma Baijiu, designated as N, was purchased at a local market with the ethanol concentration of 61.8% ABV. As shown in Figure 6, a total of five dilutions of the Baijiu sample were prepared, including four dilutions with ultrapure water to 50% ABV (N-50), 40% ABV (N-40), 30% ABV (N-30), and 20% ABV (N-20), as well as a dilution (N-30E) with an aqueous 61.8% ABV solution using the same dilution ratio as N-30. The aqueous 61.8% solution was prepared by diluting ethanol with ultrapure water. All samples were prepared fresh (no more than 24 h) before the test and stored at 26 °C.

Attribute references were prepared prior to evaluation (within 24 h) and placed into 30 mL lidded plastic cups, labeled with the reference identity. The detailed list, including all attributes, definitions, references, and reference scores, is shown in Table 1.

### 4.3. Descriptive Analysis

#### 4.3.1. Panel

A total of ten panelists (four males and six females, aged 22–35 years) from Jiangnan University were recruited as candidates for the descriptive analysis panel. The panelists were selected based on their health status, interest, availability, and experience with tasting Baijiu, as assessed through an initial test.

#### 4.3.2. Training

On the first day of training, the panelists learned about the concept of the DA method used in the study [34]. During the first six sessions (1 h each) of the training, a dilution series of samples labeled with random codes was provided to help generate aroma terms and references. A list of terms was collected from previous research to aid in the generation of aroma terms [35]. Based on the group discussion, the panel confirmed the terms, defined each term precisely, and determined appropriate reference (Table 1).

Then, panelists spent two sessions (1 h each) determining the reference intensities of each term. The references were scaled using a 15-point scale. Panelists spent nine sessions practicing the scoring of Baijiu samples, using the references as anchor points for the scale to ensure panel uniformity. The scores from the training sessions were shown to the panelists so that they could identify and correct any attributes they had rated inconsistently compared to the other panelists. The performance of the panel was assessed using R Studio to evaluate the repeatability, stability, and consistency.

#### 4.3.3. Sample Evaluation

The panelists smelled all Baijiu samples over three sessions (1 h each), with the samples presented in triplicates. A 10 mL sample was served in standard Baijiu tasting glasses, covered with glass petri dishes, and coded with random three-digit codes. The sample order was randomized for the panel. The tests were carried out in an individual booth maintained at 26 °C. The references were provided to all panelists upon arrival, and they were encouraged to smell all references before the test. Panelists were free to reevaluate any reference at any time during the test.

### 4.4. Threshold Determination

Olfactory thresholds of 40 aroma compounds in aqueous solutions of 60%, 50%, 40%, 30%, and 20% ABV were measured by 25 panelists. Panelists were informed about the nature of the compounds, and standard solutions were presented to them before the test. The panelists performed 3-AFC tests [36]. Each compound in each alcohol concentration level comprised nine forced-choice tests, with increasing concentrations differentiated by a factor of 3.0. Each test included one sample with increasing concentrations of the compound and two blank aqueous ethanol solutions. The initial concentrations of compounds were determined by the pre-test. The results of the 3-AFC tests were statistically analyzed using the best estimate threshold (BET) [37].

### 4.5. Statistical Analysis

R Studio was employed to evaluate the performance of the panel. Principle component analysis (PCA) biplot was conducted using XLSTAT software (version 2014). Column chart, scatter plots, and Spearman correlation heatmap were visualized with Origin2022b.

## Figures and Tables

**Table 1:** List of aroma attributes, definitions, references, and reference scores for the descriptive analysis.

Attribute	Definition	Reference	Reference Score
Fruity	Aroma of tropical fruits like pineapple	20 mg/L ethyl hexanoate in water	10
Ethanol	Aroma associated with ethanol	20 mg/L isoamyl alcohol in aqueous 40% ABV solution	8
Sweet	Aroma associated with honey	5 μL Le nez du vin of honey dissolved in 50 mL water	9
Jiao-aroma	The aroma characteristics of Baijiu produced by mud pit fermentation, mainly composed of ethyl hexanoate	16 mg/L propyl caproate, 32 mg/L heptanoic acid, 16 mg/L butyric acid, 3.2 mg/L ethyl caproate, 8 mg/L valeric acid, 16 mg/L acetic acid in water	7
Sour	Aroma associated with volatile acids	20 mg/L butyric acid and valeric acid in water	9
Green	Aroma associated with grass and leaves	5 μL Le nez du vin of green pepper dissolved in 50 mL water	8
Grain	Aroma associated with steamed grains, such as sorghum, rice, wheat and other grain raw materials	Steamed sorghum	8
Bran	Aroma associated with raw chaff and other accessories	Moist chaff	7
Musty	Mould-like odor	Baijiu sample with typical musty aroma	10

**Table 2:** Analysis of variance (ANOVA) F-ratios for amora attributes of Chinese strong-aroma Baijiu ^a^.

Attribute	Dilution	Panelist	Session	D-P ^b^	D-S ^b^	P-S ^b^
Fruity	6.5 ***	4.1 ***	0.8	0.9	0.3	0.8
Ethanol	19.8 ***	2.4 *	1.0	1.5	1.6	0.6
Sweet	1.7	1.8	0.3	1.1	0.5	0.9
Grain	2.9 *	2.7 **	0.2	1.3	1.2	0.9
Jiao-aroma	8.3 ***	2.6 **	0.5	1.3	1.0	0.9
Bran	1.2	2.9 **	1.0	1.1	0.8	0.4
Sour	0.3	3.4 **	0.2	1.4	0.2	0.8
Green	0.9	1.3	0.0	1.3	0.7	0.5
Musty	1.4	2.9 **	0.6	0.9	1.7	0.5

*, **, *** stand for significance at *p* < 0.05, *p* < 0.01, and *p* < 0.001, respectively. ^a^ F-ratios are shown as a source of variation. ^b^ D-P, D-S, and P-S represent the interaction between dilution sample and panelist, dilution samples and session, and panelist and session, respectively.

**Table 3:** Thresholds of 40 compounds in 5 ethanol levels.

No.	Compound	Odor	Threshold						R^2 a^	*p*
			Unit	20% ABV	30% ABV	40% ABV	50% ABV	60% ABV		
	Esters									
1	Ethyl acetate	Pineapple, apple, fruit	mg/L	85	106	106	194	397	0.7540	0.06
2	Ethyl propionate **	Banana, fruity	mg/L	2	3	6	8	12	0.9770	0.00
3	Ethyl butyrate **	Apple, pineapple, fruity, floral	μg/L	32	39	95	112	174	0.9440	0.01
4	Ethyl valerate *	Peachy, fruity, floral, sweet	μg/L	5	7	19	67	74	0.8770	0.02
5	Ethyl hexanoate	Sweet, fruity, cucumber	μg/L	27	52	65	270	687	0.7630	0.05
6	Ethyl heptanoate **	Floral, fruity, honey, sweet	μg/L	125	243	574	971	1643	0.9340	0.01
7	Ethyl caprylate *	Pear, lychee, fruity, sweet, lily	μg/L	25	54	94	256	307	0.9190	0.01
8	Ethyl nonanoate *	Ester, honey, fruity	μg/L	105	50	634	1639	2103	0.9130	0.01
9	Ethyl caprate *	Pineapple, fruity, floral	mg/L	1	1	2	3	3	0.8720	0.02
	Acids									
10	Acetic acid *	Sour	mg/L	106	256	336	336	522	0.9170	0.01
11	Propionic acid *	Sour	mg/L	1	3	5	11	26	0.8350	0.03
12	Butyric Acid	Sweat, sour, cellar mud	mg/L	1	1	1	3	2	0.6570	0.10
13	Valeric acid *	sweat, sour	μg/L	270	370	950	950	2700	0.7770	0.05
14	Hexanoic acid **	Sweat, animal, sour, sweet, fruit	mg/L	1	3	6	15	17	0.9410	0.01
15	Heptanoic acid **	Sour, sweaty, muddy, mildew	mg/L	3	12	23	27	45	0.9690	0.00
16	Octanoic acid *	Fruity, floral, greasy	mg/L	2	3	4	24	34	0.8290	0.03
17	Decanoic acid **	Goat, rubber, paint smells, animal	mg/L	1	2	6	7	8	0.9330	0.01
	Alcohols									
18	1-Propanol *	Fruity, floral, grassy	mg/L	175	228	253	888	935	0.8189	0.04
19	1-Butanol *	Fruity aroma	mg/L	140	182	263	748	972	0.8821	0.02
20	2-Butanol *	Sweet apricots	mg/L	23	20	45	93	93	0.8646	0.01
21	1-Pentanol	Sweet, mellow	mg/L	20	203	377	1188	850	0.7559	0.08
22	2-Methyl-1-butanol *	Onion, mellow	mg/L	8	18	27	65	99	0.9166	0.03
23	3-Methyl-1-butanol *	Fruity, floral, smelly	mg/L	87	151	405	782	663	0.8511	0.06
24	1-Hexanol	Mellow, sweet, green	mg/L	2	4	15	40	26	0.7007	0.02
25	Linalool *	Citrus, floral, sweet, woody	μg/L	2	11	14	98	93	0.7935	0.04
	Aldehydes and ketones									
26	Damascenone	Fruity, sweet, rose	ng/L	40	80	400	4380	3370	0.7050	0.08
27	1-Nonanal	Soap, grass, watery smell	μg/L	2	22	22	324	250	0.6950	0.08
28	β-Lonone **	raspberry, floral, violet	μg/L	0.3	2.7	7.3	15.1	15.1	0.9350	0.01
29	FEMA 3377 *	Raw greens, cucumbers, violets	ng/L	40	60	200	490	470	0.8810	0.02
	Lactones									
30	γ-Dodecalactone	Fruity, honey, creamy	μg/L	28	76	50	175	142	0.6900	0.08
31	γ-Decalactone *	Fruity, sweet, floral	μg/L	10	18	26	31	58	0.8920	0.02
32	γ-Valerolactone *	Sweet, herbaceous, smoky, cocoa	mg/L	5	15	13	277	492	0.8020	0.04
33	γ-Octanoic lactone *	Creamy, coconut creamy	μg/L	68	148	203	468	924	0.8520	0.03
34	γ-Nonanolactone *	Creamy, coconut	μg/L	13	33	60	60	156	0.8150	0.04
	Sulfur compounds									
34	Furfuryl mercaptan *	Coffee, roasted	ng/L	0.5	0.2	1.2	4.1	5.7	0.8600	0.02
35	Dimethyl trisulfide	Ether, cabbage, pickles	ng/L	58	46	72	118	241	0.7550	0.06
36	3-(Methylthio) Propionaldehyde *	musty, potatoes, vegetables	ng/L	340	60	1330	2130	4420	0.8540	0.03
37	Bis-(2-methyl-3-furyl)-Disulfide *	Toasted, onion, sulfur	ng/L	0.3	2.0	26.4	87.9	237.4	0.7900	0.04
	Others									
39	(±)-Geosmin	Earthy smell	ng/L	20	320	750	1480	6400	0.6990	0.08
40	4-Ethyl-2-methoxyphenol *	fruity, sweet, floral, smoky, rubber	μg/L	21	48	63	83	190	0.8250	0.03

^a^ Pearson correlation analysis was performed between compound threshold and ethanol concentration. *, **, represent *p* < 0.05, *p* < 0.01, respectively.

**Table 4:** The change of compound OAV during dilution.

Compounds	Content (μg/L) in 50% ABV	OAV				
		20% ABV	30% ABV	40% ABV	50% ABV	60% ABV
Esters						
Ethyl acetate	1,112,598.5	5.2	6.3	8.4	5.7	3.4
Ethyl propionate	15,056.9	3.0	3.0	2.0	1.9	1.5
Ethyl butyrate	92,752.4	1159.4	1427.0	781.1	828.1	639.7
Ethyl valerate	12,481.3	998.5	1069.8	525.5	186.3	202.4
Ethyl hexanoate	1,900,106.1	28,149.7	21,924.3	23,385.9	7037.4	3319.0
Ethyl heptanoate	7197.9	23.0	17.8	10.0	7.4	5.3
Ethyl caprylate	1387.6	22.2	15.4	11.8	5.4	5.4
Ethyl nonanoate	740.3	2.8	8.9	0.9	0.5	0.4
Ethyl caprate	2648.4	1.1	1.6	1.1	0.9	1.1
Acids						
Acetic acid	1,278,619.9	4.8	3.0	3.0	3.8	2.9
Propionic acid	14,620.7	5.8	2.9	2.3	1.3	0.7
Butyric Acid	70,147.3	28.1	42.1	56.1	23.4	42.1
Valeric acid	76,019.7	112.6	123.3	64.0	80.0	33.8
Hexanoic acid	1,900,106.1	760.0	380.0	253.3	126.7	134.1
Heptanoic acid	28,523.6	3.8	1.4	1.0	1.1	0.8
Octanoic acid	10,898.4	2.2	2.2	2.2	0.5	0.4
Decanoic acid	2143.3	0.9	0.6	0.3	0.3	0.3
Alcohols						
1-Propanol	541,850.8	1.2	1.4	1.7	0.6	0.7
1-Butanol	322,281.7	0.9	1.1	1.0	0.4	0.4
2-Butanol	55,944.9	1.0	1.7	1.0	0.6	0.7
1-Pentanol	29,225.5	0.6	0.1	0.1	0.0	0.0
2-Methyl-1-butanol	39,633.2	2.0	1.3	1.2	0.6	0.5
3-Methyl-1-butanol	218,340.1	1.0	0.9	0.4	0.3	0.4
1-Hexanol	134,411.9	26.9	20.2	7.2	3.4	6.2
Linalool	15.4	3.1	0.8	0.9	0.2	0.2
Aldehydes and ketones						
Damascenone	14.3	142.6	107.0	28.5	3.3	5.1
1-Nonanal	672.1	134.4	18.3	24.4	2.1	3.2
β-Lonone	0.4	0.6	0.1	0.0	0.0	0.0
FEMA 3377	8.5	85.1	85.1	34.0	17.4	21.7
Lactones						
γ-Dodecalactone	2.9	0.0	0.0	0.0	0.0	0.0
γ-Decalactone	30.7	1.2	1.0	0.9	1.0	0.6
γ-Nonanolactone	134.0	4.1	2.4	1.8	2.2	1.0
Sulfur compounds						
Furfuryl mercaptan	37.5	30,005.8	112,521.6	25,004.8	9148.1	7896.3
Dimethyl trisulfide	50.4	347.4	657.0	559.7	426.9	250.8
3-(Methylthio) propionaldehyde	414.8	488.0	4147.8	249.5	194.7	112.6
Others						
(±)-GEOSMIN	12.3	246.1	23.1	13.1	8.3	2.3
4-Ethyl-2-methoxyphenol	76.5	1.5	1.0	1.0	0.9	0.5

## Data Availability

All experimental data acquired are reported in the manuscript.

## Supplementary Materials

The following supporting information can be downloaded at: https://www.mdpi.com/article/10.3390/molecules30040933/s1, Table S1: Mean intensity rating for Baijiu samples; Table S2: Thresholds ratio of compounds in 5 ethanol levels; Table S3: The change of concentration, threshold, OAV and OAV ratio in Baijiu during dilution.
