# Supplementary material for: The Effect of Ethanol on the Compound Thresholds and Aroma Perception in Chinese Baijiu
Source: Molecules. 2025 Feb 17;30(4):933. doi: 10.3390/molecules30040933 (PMC11858315; doi:10.3390/molecules30040933)
Supplement: Supplementary file 1 [file molecules-30-00933-s001.zip › molecules-3348960-supplementary.pdf]

Table S1 Mean intensity rating for Baijiu samples

Attribute	N	N-50	N-40	N-30	N-20	N-30E
Fruity	6.80 ^{c}	8.15 ^{a}	8.43 ^{a}	7.87 ^{ab}	6.27 ^{c}	7.15 ^{bc}
Ethanol	9.40 ^{a}	7.80 ^{b}	8.70 ^{ab}	6.78 ^{c}	5.72 ^{d}	8.78 ^{a}
Jiao-aroma	7.92 ^{bc}	8.85 ^{ab}	9.55 ^{a}	8.53 ^{b}	6.80 ^{d}	7.33 ^{cd}
Sweet	7.80 ^{a}	7.67 ^{a}	7.88 ^{a}	7.33 ^{ab}	6.67 ^{b}	7.33 ^{ab}
Grain	7.23 ^{abc}	7.03 ^{bc}	7.53 ^{ab}	7.62 ^{ab}	8.20 ^{a}	6.42 ^{c}
Bran	7.80 ^{a}	7.37 ^{ab}	7.50 ^{ab}	7.68 ^{a}	7.31 ^{ab}	6.68 ^{b}
Sour	7.05 ^{a}	7.23 ^{a}	7.32 ^{a}	7.02 ^{a}	7.08 ^{a}	6.88 ^{a}
Green	6.05 ^{a}	6.03 ^{a}	6.37 ^{a}	5.88 ^{a}	5.53 ^{a}	5.60 ^{a}
Musty	6.97 ^{ab}	6.27 ^{b}	6.85 ^{ab}	6.93 ^{ab}	7.35 ^{a}	6.35 ^{b}

Superscripts of the same letter within an attribute indicate no significant difference by Fisher's least significant difference (LSD) test at $\alpha = 0.05$.

Table S2 Thresholds ratio of compounds in 5 ethanol levels

No.	Compound	CAS	Threshold Ratio (Take the threshold in 20% ABV as 1.0)				
			20% ABV	30% ABV	40% ABV	50% ABV	60% ABV
	**Esters**						
1	Ethyl acetate	141-78-6	1.0	1.2	1.2	2.3	4.7
2	Ethyl propionate	105-37-3	1.0	1.5	3.0	3.9	5.5
3	Ethyl butyrate	105-54-4	1.0	1.2	3.0	3.5	5.5
4	Ethyl valerate	539-82-2	1.0	1.4	4.0	14.1	15.6
5	Ethyl hexanoate	123-66-0	1.0	1.9	2.4	10.0	25.6
6	Ethyl heptanoate	106-30-9	1.0	2.0	4.6	7.8	13.2
7	Ethyl caprylate	106-32-1	1.0	2.2	3.8	10.3	12.4
8	Ethyl nonanoate	123-29-5	1.0	0.5	6.0	15.6	20.0
9	Ethyl caprate	110-38-3	1.0	2.1	3.6	5.9	5.1
	**Acids**						
10	Acetic acid	64-19-7	1.0	2.4	3.2	3.2	4.9
11	Propionic acid	79-09-4	1.0	2.4	4.4	9.0	20.5
12	Butyric Acid	107-92-6	1.0	1.1	0.9	2.5	2.3
13	Valeric acid	109-52-4	1.0	1.4	3.5	3.5	10.0
14	Hexanoic acid	142-62-1	1.0	2.7	5.8	14.0	16.5
15	Heptanoic acid	111-14-8	1.0	4.4	8.5	10.0	16.5
16	Octanoic acid	124-07-2	1.0	1.3	1.8	12.5	17.4
17	Decanoic acid	334-48-5	1.0	2.8	8.1	9.0	10.6
	**Alcohols**						
18	1-Propanol	71-23-8	1.0	1.3	1.4	5.1	5.3

19	1-Butanol	71-36-3	1.0	1.3	1.9	5.3	6.9
20	2-Butanol	78-92-2	1.0	0.9	1.9	4.0	4.0
21	1-Pentanol	71-41-0	1.0	9.9	18.4	58.0	41.5
22	2-Methyl-1-butanol	137-32-6	1.0	2.3	3.5	8.5	13.0
23	3-Methyl-1-butanol	123-51-3	1.0	1.7	4.7	9.0	7.6
24	1-Hexanol	111-27-3	1.0	2.0	7.8	21.3	13.8
25	Linalool	78-70-6	1.0	4.8	6.0	42.9	40.5
	**Aldehydes and ketones**						
26	Damascenone	23696-85-7	1.0	1.8	9.0	99.9	76.9
27	1-Nonanal	124-19-6	1.0	9.5	9.5	136.7	105.2
28	β-Lonone	79-77-6	1.0	10.0	27.0	56.2	56.2
29	FEMA 3377	557-48-2	1.0	1.6	5.6	13.7	13.0
	**Lactones**						
30	γ-Dodecalactone	2305-05-7	1.0	2.7	1.8	6.2	5.1
31	γ-Decalactone	706-14-9	1.0	1.9	2.7	3.2	5.9
32	γ-Valerolactone	108-29-2	1.0	3.2	2.8	59.2	105.3
33	γ-Octanoic lactone	104-50-7	1.0	2.2	3.0	6.9	13.7
34	γ-Nonanolactone	104-61-0	1.0	2.6	4.7	4.7	12.1
	**Sulfur compounds**						
34	Furfuryl mercaptan	98-02-2	1.0	0.3	2.3	8.1	11.2
35	Dimethyl trisulfide	3658-80-8	1.0	0.8	1.2	2.0	4.2
36	3-(Methylthio)propionaldehyde	3268-49-3	1.0	0.2	3.9	6.2	13.0
37	Bis (2-methyl-3-furyl) disulfide	28588-75-2	1.0	5.9	76.9	256.1	691.8
	**Others**						
39	(±)-Geosmin	16423-19-1	1.0	21.9	50.6	99.9	432.0

40	4-Ethyl-2-methoxyphenol	2785-89-9	1.0	2.3	3.0	3.9	9.0

Table S3 The change of concentration, threshold, OAV and OAV ratio in Baijiu during dilution

Compound	Unit	Concentration in 50%ABV	Threshold		OAV		OAV%[a]	
			30%ABV	50%ABV	30%ABV	50%ABV	30%ABV	50%ABV
Ethyl propionate	mg/L	38	3.00	8.00	7.63	4.77	8%	16%
Ethyl heptanoate	mg/L	6	0.24	0.97	14.90	6.21	15%	21%
Ethyl caprylate	mg/L	1	0.05	0.26	8.77	3.08	9%	11%
Ethyl caprate	mg/L	4	1.00	3.00	2.45	1.36	2%	5%
Propionic acid	mg/L	10	3.00	11.00	1.97	0.89	2%	3%
Butyric Acid	mg/L	10	1.00	3.00	5.70	3.17	6%	11%
Valeric acid	mg/L	2	0.37	0.95	2.45	1.59	2%	5%
Hexanoic acid	mg/L	24	3.00	15.00	4.82	1.61	5%	6%
2-Methyl-1-butanol	mg/L	69	18.00	65.00	2.31	1.07	2%	4%
1-Hexanol	mg/L	9	4.00	40.00	1.28	0.21	1%	1%
Linalool	μg/L	25	11.00	98.00	1.39	0.26	1%	1%
Damascenone	μg/L	5	0.08	4.38	37.58	1.14	38%	4%
(±)-Geosmin	μg/L	4	0.32	1.48	7.50	2.70	8%	9%
4-Ethyl-2-methoxyphenol	μg/L	80	48.00	83.00	1.00	0.97	1%	3%

[a] OAV_i% means the percentage of OAV of this compound in the total OAV.
